# Enhancement of Heat and Drought Stress Tolerance in Rice by Genetic Manipulation: A Systematic Review

**DOI:** 10.1186/s12284-022-00614-z

**Published:** 2022-12-23

**Authors:** Yingxue Yang, Jianping Yu, Qian Qian, Lianguang Shang

**Affiliations:** 1 Shenzhen Branch, Guangdong Laboratory of Lingnan Modern Agriculture, Genome Analysis Laboratory of the Ministry of Agriculture and Rural Affairs, Agricultural Genomics Institute at Shenzhen, Chinese Academy of Agricultural Sciences, Shenzhen, 518120 China; 2 College of Plant Science and Technology, Key Laboratory of New Technology in Agricultural Application, Beijing University of Agriculture, Beijing, 102206 China; 3 China National Rice Research Institute (CNRRI), Chinese Academy of Agricultural Sciences, Hangzhou, 311401 China

**Keywords:** Rice, Heat stress, Drought stress, Regulation, Tolerance

## Abstract

As a result of global warming, plants are subjected to ever-increasing abiotic stresses including heat and drought. Drought stress frequently co-occurs with heat stress as a result of water evaporation. These stressors have adverse effects on crop production, which in turn affects human food security. Rice is a major food resource grown widely in crop-producing regions throughout the world. However, increasingly common heat and drought stresses in growth regions can have negative impacts on seedling morphogenesis, reproductive organ establishment, overall yield, and quality. This review centers on responses to heat and drought stress in rice. Current knowledge of molecular regulation mechanisms is summarized. We focus on approaches to cope with heat and drought stress, both at the genetic level and from an agricultural practice perspective. This review establishes a basis for improving rice stress tolerance, grain quality, and yield for human benefit.

## Background

Climate change has caused increased frequency and magnitude of hot and dry spells (Gornall et al. 2010). The global average temperature is predicted to surge by 7 °C by the end of this century (Solomon et al. 2007). This will have great impacts on the productivity of important crops and impair normal ecosystem functions. For example, temperature increases of 3–4 °C have been shown to reduce crop yield by 15–35% in Africa, Asia, and the Middle East (Gregory et al. 2005). The evident constraints can further accelerate the competition for environmental resources caused by human population growth. The growing demand for food necessitates advanced research in crop improvements to meet the challenge of climate change (Dhankher and Foyer 2018).

Rice (*Oryza sativa* L.) is an important global food resource, serving half of the world’s population (Gnanamanickam 2009; Shang et al. 2022). With intensification of the global greenhouse effect, heat stress has become one of the main obstacles to consistent rice production; any increase in temperature beyond the optimum range can decrease rice production when is the plants themselves are in a vulnerable stage (Fahad et al. 2019). For example, in 2003, rice fields in southern China experienced rare high temperature conditions. In some fields, temperatures above 38 °C lasted for 20 days from July to August, causing great losses to rice production (Xia and Qi 2004). High temperature stress is predicted to decrease the world’s rice yield by 40% in the next century due to the negative effects it has during multiple developmental stages including early floral meristem growth, gametogenesis, flowering, and grain filling (Jagadish et al. 2013). Thus, studying the mechanisms of and countermeasures to high temperature damage in rice is of great significance in global food security.

Plant agriculture provides the basis for many nations’ economies and food supplies. However, agricultural practices consume an enormous amount of fresh water, and agricultural output worldwide consistently suffers from water deficits due to global warming, decreases in rainfall, and increased frequency of dry spells. Strategies for water saving in agriculture are therefore of great significance. Drought stress affects plant performance by influencing morphology, leading to overall decreased growth, leaf area reduction, and early maturity. Physiological changes include closure of stomata and increased oxidative stress. There are different approaches to manage the effects of water scarcity. Conventional breeding for yield improvement has long been used. Marker assisted selection (MAS) is the main method of genomics-assisted breeding used to improve water use efficiency, stomatal conductance, and osmotic adjustments.

## Heat Stress

Rice originated in tropical or subtropical regions and therefore thrives in relatively warm temperatures and high humidity environments (De Datta 1981). However, during the flowering period, continuous high temperatures prevent the spikelet from absorbing water normally, and pollen vitality is reduced due to the lack of water (Khatun et al. 1995). The germination rate decreases, pollen tube elongation stops, and the fertilization process cannot form pellets, ultimately resulting in decreased yield (Barnabás et al. 2008).

## The Influence of Heat Stress on Rice

### The Influence of Heat Stress on Rice Production and Quality

Rice requires suitable temperatures at every stage of growth and development for survival; adverse effects occur if the temperature is beyond the maximum of the optimal range (Fahad et al. 2016b). Responses to heat stress differ between stages of growth and development. In the seedling stages, heat-stressed rice often exhibits delayed growth, reduced tiller number, and changed leaf color. The most sensitive stages to heat stress are the booting, heading, and flowering stages (Wang et al. 2019). Heat stress can reduce the number of spikelets per panicle by causing attenuated differentiation of secondary branches and florets during the panicle-initiation stage. Spikelet sterility is caused by reduced functional female and male organs as a result of high temperature during the flowering stage (Fábián et al. 2019), further affecting the seed setting rate and yield. Heat stress has the largest impact during the days before flowering, peaking on the flowering day. Tolerance to heat stress is distinct between heat-tolerant varieties and heat-sensitive varieties. The anther cracking coefficient, number of pollen grains on the stigma, pollen vigor, and stigma vigor of heat-tolerant varieties have higher values than those of heat-sensitive varieties. This demonstrates that heat-tolerant varieties display a decreased influence of heat stress on the seed setting rate and yield.

Reduction in quality is another common effect of high temperatures, although this is constrained by the genetic factors of each rice variety. Short-term heat stress (above 35 °C) at the booting stage reduces panicle size, milling characteristics, and amylose content (Zhen et al. 2019). Rice grain weight is determined by grain volume and grain plumpness, both of which can be severely affected by high temperature (Wang et al. 2016b). Two *indica* rice varieties, Shuanggui 1 (heat sensitive) and Huanghuazhan (heat tolerant), have demonstrated significantly reduced 1000-grain weight and grain yield in response to heat stress, an effect that was greater in the heat-sensitive than in the heat-tolerant variety (Cao et al. 2008). An average daily temperature higher than 27 °C during 20 days before heading was shown to be a vital threshold in inducing chalky grains in Japanese cultivars (Wakamatsu 2010). Grain quality plays an important role in the economic value of rice; grain chalkiness may greatly reduce the value due to grain breakage during milling and customers’ preferences for clearer grains (Lyman et al. 2013). Modest (1 °C) increases in daily maximum and minimum temperatures can reduce paddy yield by up to 10% while increasing chalkiness. Milling outcomes determine the edible rice yield and therefore have a large impact on global human nutrition. Investigation of both rice yield and quality in response to heat stress is thus an important issue (Jagadish et al. 2015).

### The Influence of Heat Stress on Rice Physiology

Photosynthesis is the major determinant of rice production and is one of the most sensitive processes to high temperature (Berry and Bjorkman 1980). Various aspects of photosynthesis can be adversely affected by heat stress, ultimately decreasing yield. These include light energy capture, photosystem II (PSII)- and photosystem I (PSI)-mediated electron transfer, and carbon assimilation processes (Stasik and Jones 2007). High temperature can decrease leaf photosynthetic rate, stomatal conductance, and chlorophyll content. Chlorophyll fluorescence is widely used as a proxy to evaluate impairment of the leaf photosynthetic machinery, including structural and functional changes in response to heat stress (Mathur et al. 2011). PSII contains the oxygen-evolving complex (OEC), which is considered the most heat-sensitive component of the photosynthetic machinery, and can be affected by a variety of stress factors such as drought, salinity, low and high temperatures, and UV radiation. The severity of stress-induced damage relies on the balance between damage and repair systems (Murata et al. 2007). High temperature induces a large amount of activated Rubisco expressed in rice to alleviate heat stress inhibition of Rubisco enzymes and maintain the photosynthetic rate within a certain range (Scafaro et al. 2010). Heat stress affects photosynthesis by two basic mechanisms: direct damage of photosynthesis apparatus and reduction of protein synthesis due to the presence of reactive oxygen species (ROS). ROS scavenging has been reported to diminish the damage induced by heat stress (Suzuki and Mittler 2006).

As signaling molecules, ROS play a key role in regulating biological processes such as growth, development, and stress responses (Miller et al. 2008). Under stresses such as high temperature, ROS accumulation causes oxidative damage to cells and inhibits photosynthesis. Specifically, ROS including singlet oxygen (^1^O_2_), superoxide anion radicals (O^2−^), hydrogen peroxide (H_2_O_2_), and hydroxyl radicals (OH^−^) accumulate in the mitochondria, endoplasmic reticulum, and peroxisomes. This can result in lipid peroxidation, leading to the destruction of cell membrane structure, increased plasma membrane permeability, and reduced photosynthetic rate (Pospíšil 2016).

To prevent plant damage, active oxygen is neutralized by the antioxidant mechanism, which can be divided into enzymatic and non-enzymatic systems (Sankhalkar and Sharma 2002). Enzymatic systems primarily include superoxide dismutase (SOD), glutathione reductase (GR), peroxidase (POD), and catalase (CAT). The non-enzymatic system comprises anti-ascorbic acid (AsA) and reduced glutathione (GSH). The anti-oxidation system can also be affected by stress; antioxidant enzymes are sensitive to temperature, and activation occurs in different temperature ranges. The activity of antioxidant enzymes also varies between different crops, varieties, growth stages, and growing seasons (Almeselmani et al. 2006).

Plant endogenous hormones play a key role in rice growth and yield formation. High temperature can induce changes in rice endogenous hormone levels during grain filling, which in turn affects yield (Wu et al. 2019). The transport and degradation of rice cytokinin under high temperature stress is closely related to a decrease of cytokinin expression in the rice panicle and a decrease in panicle grain number (Wu et al. 2017). Salicylic acid (SA) can reduce damage to rice and increase the grain yield under heat stress by preventing spikelet degeneration and enhancing spikelet number per panicle (Zhang et al. 2017). Abscisic acid (ABA) degradation is strongly related to increasing temperature during seed germination in rice (Wu and Hong 2021). Heat stress lowers levels of indoleacetic acid (IAA) by promoting the formation of IAA–amino acid conjugates and decreasing expression of genes in the IAA biosynthetic pathway. Gibberellic acid (GA) content is also decreased in young rice panicles under high temperatures (Wu et al. 2016). Grain weight of rice varieties decreases with changes in endosperm levels of Cytokinin (CTK), IAA, and ABA induced by heat stress (Wu et al. 2016). Phytohormone biosynthesis and transport determine the levels of phytohormones associated with rice grain yield under high temperature.

### Heat-Related Genes and Regulation Mechanisms

Stress causes plants to change their physiological, biochemical, molecular, and cellular parameters to adapt to the unfavorable living environment (Lamaoui et al. 2018). Identifying the link between key functional genes and stress resistance will aid in understanding the damage mechanisms of stressors and the molecular mechanisms of plant adaptation to adversity at the transcriptional level. At present, a large number of high temperature-related genes have been cloned in rice (Table 1), and they can be roughly divided into five functional categories: heat shock proteins, heat shock transcription factors, stress-related transcription factors, enzymes, and other proteins.

**Table 1:** Genes involved in heat stress

Category	Gene	Accession number	Gene product	Functional role	References
Stress-related transcription factors	*OsAREB1*	LOC_Os06g10880	bZIP transcription factor	Regulates the expression of abiotic stress-responsive genes through an ABA-dependent pathway	(Hossain et al. 2010)
	*OsMYB55*	LOC_Os05g48010	R2R3-MYB transcription factor	Increases amino acid metabolism, improving high temperature tolerance	(El-Kereamy et al. 2012)
	*OsNTL3*	LOC_Os01g15640	NAC transcription factor	Interacts with OsbZIP74 and plays an important role in thermotolerance	(Liu et al. 2020)
Heat shock transcription factors	*OsHsfA2c*	LOC_Os10g28340	Heat shock transcription factor	Involved in transcriptional regulation of rice cytoplasmic gene HSP100	(Singh et al. 2012)
	*OsHSF7*	LOC_Os03g06630	Heat shock transcription factor	Up-regulates Hsps and other protective genes during heat treatment, conferring higher basic heat tolerance	(Liu et al. 2009)
Heat shock proteins	*HSP101*	LOC_Os05g44340	Heat shock protein	Post-transcriptional interactions of HSA32/HSP101 occur in heat-treated rice seedlings, prolonging the effect of heat training	(Lin et al. 2014)
Enzymes	*GAD3*	LOC_Os03g13300	Glutamate decarboxylase	Involved in tolerance to high temperature	(El-Kereamy et al. 2012)
	*OsHCI1*	LOC_Os10g30850	RING finger E3 ligase	Drives the nuclear export of multiple substrate proteins, and its heterologous	(Lim et al. 2013)
	*OsHTAS*	LOC_Os09g15430	RING Finger Ubiquitin E3 Ligase	Enhances rice heat tolerance by mediating H_2_O_2_-induced stomata closure	(Liu et al. 2016b)
	*TCM5*	LOC_Os05g34460	Deg Protease Protein	Has a key role in chloroplast development and PSII functional maintenance under high temperature	(Zheng et al. 2016)
	*EG1*	LOC_Os01g67430	Lipase	Promotes homeostasis of floral organs and tolerance of temperature fluctuations through high temperature-mediated mitochondrial lipase pathway	(Zhang et al. 2016)
	*OsTT1*	LOC_Os03g26970	α2 subunit of the 26 S proteasome	Degrades toxic denatured proteins and maintains high temperature response process	(Li et al. 2015b)
	*TOGR1*	LOC_Os03g46610	DEAD-box RNA helicase	Involved in normal rRNA precursor processing under high temperature conditions and is a chaperone protein of the nucleolar SSU complex; important for cell proliferation and growth under high temperature	(Wang et al. 2016a)
	*OsNSUN2*	LOC_Os09g29630	NOP2/Sun (NSUN) RNA methyltransferase	Regulates the 5-methylcytosine (m5C) mRNA modification, increasing the efficiency of mRNA translation and maintaining normal growth at higher temperatures	(Tang et al. 2020)
	*OsHES1*	LOC_Os08g10600	UDP-N-acetylglucosamine pyrophosphorylase	Plays a key role in adaptation to high-temperature stress and in the maintenance of chloroplast function	(Xia et al. 2021)
	*OsAET1*	LOC_Os05g45890	tRNA^His^ guanylyltransferase	Regulates the high temperature response by playing a dual role in tRNA modification and translational control	(Chen et al. 2019)
	*OsTT3.1*	LOC_Os03g49900	E3 ligase	TT3.1 ubiquitinates chloroplast precursor protein TT3.2 for vacuolar degradation, TT3.1 might serve as a potential thermosensor	(Zhang et al. 2022)
Other proteins	*OsTT3.2*	LOC_Os03g49940	Chloroplast precursor protein	Mature TT3.2 proteins in chloroplasts are essential for protecting thylakoids from heat stress	(Zhang et al. 2022)
	*SLG1*	LOC_Os12g39840	Cytosolic tRNA 2-thiolation protein 2	Regulates the level of tRNA thiolation, positively regulating heat tolerance	(Xu et al. 2020)
	*OsLEA5*	LOC_Os05g50710	Late embryogenesis abundant protein	Maintains the stability of LDH under heat stress	(He et al. 2012)
	*HSA32*	LOC_Os06g46900	Heat stress-associated 32-KD protein	Post-transcriptional interactions of HSA32/HSP101 occur in heat-treated rice seedlings, prolonging the effect of heat training	(Lin et al. 2014)
	*OsCNGC16*	LOC_Os05g42250	Cyclic nucleotide-gated ion channel protein	Mutation can significantly reduce or eliminate cytoplasmic calcium influx induced by temperature stress	(Cui et al. 2020)
	*OsTT2*	LOC_Os03g29380	Gγ subunit	TT2 controls rice thermotolerance through SCT1-dependent alteration of wax biosynthesis	(Kan et al. 2022)
	*OsANN1*	LOC_Os02g51750	Calcium-binding protein	Improves SOD and CAT activity and regulates H_2_O_2_ content and redox balance, providing comprehensive cell protection against abiotic stress	(Qiao et al. 2015)

Heat shock proteins (Hsps) are highly abundant in plants, constituting one of the main molecular chaperone protein types identified to date. They are typically induced upon exposure to high temperature, helping specific proteins to fold correctly and assisting in transmembrane transfer during plant stress responses. Hsps therefore have great biological significance in enhancing plant stress tolerance by preventing irreversible aggregation of denatured proteins. A number of studies have focused on identification and functional elucidation of rice heat shock proteins (Wang et al. 2014), which can be divided into two subgroups based on the molecular mass. High molecular mass Hsps include Hsp70, Hsp90, and Hsp100, and small heat shock proteins (sHsps) include Hsp20. Overexpression of *OsHSP18.6* is known to increase thermotolerance (Wang et al. 2008), and overexpression of sHSP17.7 enhances rice tolerance to heat and UV-B exposure (Murakami et al. 2004). These findings indicate that Hsps are important elements in heat response regulation, although the molecular pathways related to Hsp expression are not clearly understood.

Heat shock factors (Hsfs) can activate Hsps at the transcriptional level (Hu et al. 2009). During heat stress, Hsfs are activated as the terminal components of a signal transduction chain and mediate expression of heat shock genes by binding to heat shock element (HSE) (Wu 1995). The Hsf family is much larger in plants than in other types of eukaryotes, comprising more than 20 members in plants. An amino acid change in HsfA4b causes a lesion-mimic phenotype in rice leaf in response to heat stress (Yamanouchi et al. 2002). OsHsfB4b is primarily involved in the rice heat stress response, as evidenced by up-regulation of *OsHsfB4b* following heat stress and the polymeric interactions it forms with various OsHsfs (Mittal et al. 2011). Recent findings have suggested that there is crosstalk between Hsfs, Hsps, and ROS under heat stress. High temperature can trigger Hsf activation directly or indirectly via ROS. Hsfs promote Hsp expression and prevent subsequent oxidative damage by stimulating ROS scavenger gene expression (Driedonks et al. 2015). OsHsfA2d encodes two isoforms, one of which functions under normal temperatures; in response to heat stress, OsHSFA2d is alternatively spliced into a transcriptionally active form, OsHSFA2dI, which may help cells to establish protein folding balance by participation in the unfolded protein response (Cheng et al. 2015).

In addition to Hsfs, other transcription factors containing stress elements have also been found to be involved in heat stress. The most well-studied transcription factor types include bZIPs, MYB/MYC, WRKY, AP2/EREBP, and NAC. Their structure is usually composed of DNA binding domains, transcription activation domains, oligomerization sites, and nuclear localization signals. bZIP transcription factors are widely distributed and conserved among eukaryotes, and improve tolerance to various environmental stresses through the endoplasmic reticulum (ER) signaling pathway. MYB factors represent a family of proteins containing a conserved MYB DNA binding domain and may regulate plant heat tolerance by participating in calcium signaling pathways (Li et al. 2015a). Overexpression of OsMYB55 in rice leads to a higher total amino acid content, enhancing heat tolerance in the vegetative stage (El-Kereamy et al. 2012). Overexpression of *OsWRKY11* under the control of the HSP101 promoter significantly improves the high temperature and drought resistance of transgenic rice seedlings (Wu et al. 2009). Dehydration response element binding proteins (DREBs) are a subfamily within the AP2/CBF transcription factor family. Most studies have indicated that DREB2A primarily regulates the heat stress response by large-scale induction of HsfA3 expression. Overexpression of OsNAC3 in rice enhances tolerance to heat, drought, and oxidative stress by regulating ROS homeostasis, whereas suppression of OsNAC3 enhances stress sensitivity (Fang et al. 2015).

In recent years, a large number of enzymes involved in multiple biological pathways have been reported to participate in the rice heat stress response. The thermo-tolerance 1 gene (*TT1*) for quantitative heat resistance traits in rice was successfully identified, and revealed a new mechanism of heat resistance in crops (Li et al. 2015b). *TT1* can enhance the heat resistance of a variety of plants including rice, turfgrass, and cruciferae. The heat-sensitive dwarf mutant *togr1-1* was obtained by natural mutation of indica rice. Map-based cloning showed that *TOGR1* encodes a temperature-dependent DEAD-box RNA lyase that contains nine protein motifs. It is involved in normal processing of pre-rRNA under high temperature conditions. Mutations in the *TOGR1* gene can prevent TOGR1 from being recruited to form a functional pre-rRNA processing body, hindering the biosynthesis of rRNA. Upregulating *TOGR1* can effectively improve the heat tolerance of rice plants (Wang et al. 2016a). *OsHCI1* encodes a ring finger E3 ligase, which accumulates in the nucleus at high temperatures and mediates the ubiquitination of nuclear substrates prior to export. Heterologous expression of *OsHCI1* in *Arabidopsis thaliana* can significantly enhance its high temperature tolerance (Lim et al. 2013). *OsAPX2* is a rice cytoplasmic ascorbate peroxidase gene that is involved in the expression of ascorbate peroxidase under heat stress at the seedling stage (Chou et al. 2012).

As a large number of heat-related genes have been cloned, some key heat-tolerant signaling pathways in rice have been gradually clarified. These pathways primarily include post-transcriptional regulation, the ubiquitin proteasome pathway, metabolic pathways, and the calcium ion signaling pathway. Transcript modification has important implications for environmental temperature perception and response to heat stress. Several heat tolerance-related genes associated with post-transcriptional regulation have been reported in rice in recent years. For example, *OsNSUN2* mediates methylation of photosynthesis-related detoxification gene mRNAs in the nucleus at high temperatures; this improves translation efficiency and ensures normal growth (Tang et al. 2020). The accumulation of some metabolites has a significant contribution to the process of high temperature tolerance. The lipase gene *EG1*, localized in the mitochondria and plastids, mediates the mitochondrial lipase pathway under high temperature, regulating lipid metabolism and downstream gene expression (Zhang et al. 2016). High cytoplasmic levels of Ca^2+^ promote heat tolerance in plants. These pathways form a complex regulatory network that is important for rice survival, development, and reproduction in dynamic temperature conditions.

## Drought Stress

As a result of industrialization, agricultural development, human population growth, and global temperature increases, water shortages will become increasingly common and severe, restricting rice production (Aide 2019). Rice has different water requirements at different growth stages, and the responses to degrees of drought stress at each stage thus also differ. Suffering from drought stress in particularly sensitive stages will significantly inhibit the growth, development, and physiological characteristics of rice, leading to poor quality and a substantial decline in yield. Therefore, understanding the mechanistic effects of drought stress on rice will contribute to better utilization of water resources in rice production.

### The Influence of Drought Stress on Rice

#### The Influence of Drought Stress on Rice Growth

Water deficits affect rice growth and development at all stages (Boonjung et al. 1996). Drought stress impacts the leaf area index, plant height, root length, and effective panicle number (Alou et al. 2018). Leaf rolling is the initial symptom of drought stress, limiting water loss by reducing leaf area (Ji et al. 2012). Severe drought reduces leaf area by inhibiting the expansion and division of mesophyll cells, leading to a decline in photosynthesis and a decrease in material accumulation (Davatgar et al. 2012). Drought stress also has a significant effect on plant height (Anjum et al. 2017). At the early stage of panicle differentiation, water control treatment increases rice plant height, which provides a certain growth compensation. Severe drought stress in the mid-tillering stage significantly reduces plant height. Roots are critical structures for providing water and nutrients to allow plant growth. Under drought stress, rice roots increase in hair length and density, which is an instinctive response of the plant. In the early stage of drought stress, root growth is accelerated to promote water absorption by inducing many proteins to participate in root morphogenesis and carbon/nitrogen metabolism (Jaleel et al. 2009). The reproductive growth stage of rice is highly sensitive to drought stress. Drought hinders the development of rice reproductive organs, reducing the number of fertile pollen grains, prolonging the flowering period, and causing abnormal anther cracking. During the middle and late stages of grouting, drought leads to premature leaf senescence, shortened grouting duration, limited supply of assimilates, and reduced grain weight (Prathap et al. 2019).

#### The Influence of Drought Stress on Rice Production and Quality

Drought stress treatments at different growth stages of rice have different effects on yield, but they all result in a decrease. Drought has an impact on rice dry matter accumulation and distribution, which are the bases for yield formation (Ye et al. 2013). Drought decreases dry matter accumulation by suppressing the photosynthetic rate. Dry matter quality of various organs in different periods of short-term drought stress have decreased significantly. Drought stress occurring in different growth periods or at different severity levels have different effects on the components of yield. Drought at the tillering stage has a greater impact on the number of effective panicles per plant (Mukamuhirwa et al. 2020). Mild drought stress increases the number of effective panicles and grains and the seed setting rate, thus increasing yield (Mukamuhirwa et al. 2020). Drought stress during the flowering stage leads to a significant decrease in 1000-seed weight and seed setting rate, resulting in a decrease in per-plant yield (Wu et al. 2011). Drought stress also causes the ear number to decrease at each stage of growth, all of which show more severe effects as the level of stress is increased. Drought stress at the tillering stage has the greatest impact on effective ear number, a serious decline in which causes a significant decrease in yield. The effective panicle number is primarily affected during the tillering and panicle differentiation stages; the number of grains per panicle is most affected during the panicle differentiation and heading stages, and the seed setting rate and 1000-grain weight are most affected in the heading and seed setting stages.

Rice quality is the comprehensive result of a variety of genetic characteristics and environmental conditions, including the degree of drought stress (Krishnan et al. 2011; Gaballah et al. 2021). Moderate soil drought during the grain-setting period can significantly increase the filling rate and the activity of key enzymes in the sucrose-starch metabolic pathway in the grain, reduce the level of endogenous ethylene, significantly increase the maximum viscosity and disintegration values of rice, and reduce the chalkiness, improving overall quality; in contrast, severe drought has the opposite results (You et al. 2017).

#### The Influence of Drought Stress on Rice Physiology

Drought stress in rice causes the photosynthesis rate to decrease (Xiong et al. 2017). One reason is stomatal restriction: the stress causes the stomata to close and reduces the stomata conductance, resulting in the obstruction of CO_2_ supply, which reduces photosynthesis and material production (Lou et al. 2017). The other reason is non-stomatal restriction. Stress inhibits photosynthesis by affecting the activity of Rubisco and the PSII structure of the photosystem (Zhang et al. 2016). Chlorophyll content is one of the most widely used physiological indicators because it directly affects the efficiency of photosynthesis (Croft et al. 2017). Therefore, rice that can maintain high chlorophy II content under drought stress would be expected to have good drought resistance (Nahar et al. 2018). Drought stress resistance can also be measured by the ratio of chlorophyll a and chlorophyll b content, a higher ratio of which indicates stronger resistance to drought stress (Maisura et al. 2014).

Reactive oxygen plays an important role in regulating plant growth and development. Stressors such as drought can cause the accumulation of ROS in rice; excessive production and accumulation of ROS can destroy the membrane lipid structure. The main product of plant cell membrane lipid peroxidation is malondialdehyde (MDA), and mass fractions of MDA and free proline (Pro) have been shown to continually increase under drought stress (Yang et al. 2014). Maintaining the balance of ROS requires regulation of the antioxidant system. As discussed above related to heat stress, the main regulatory factors are SOD, POD, CAT, GR, ascorbic acid (ASA), and GSH. Water deficit increases the activity of rice SOD and CAT, which can inhibit the accumulation of MDA. Drought-tolerant rice varieties have a stronger ability than ordinary varieties to regulate the antioxidant system, and can quickly eliminate excess MDA to maintain it at a low level.

Phytohormones play a key role in regulating plant growth, development, and stress responses. Moderate soil drought during the fruiting stage has been shown to cause changes in the hormone balance of rice grains, especially the decrease of gibberellin and the increase of ABA, which promotes the storage of 14 C in the stem and sheath and accelerates the grain filling rate (Yang et al. 2001). Moderate drought treatment and re-irrigation afterwards can significantly increase the CTK content in leaves and grains, improving the photosynthetic capacity of crops, the absorption and utilization of nitrogen, proliferation of endosperm cells, and grain yield (Zhang et al. 2010; Talla et al. 2016; Zhang et al. 2012). Plants also contain a variety of polyamines (PAs), such as putrescine (Put), spermidine (Spd), and spermine (Spm). PAs play a role in regulating processes such as growth and development, morphogenesis, and adversity responses; the relative content of Spd and Spm has a significant positive correlation with the drought resistance coefficient of different rice varieties (Yang et al. 2007).

### Drought-Related Genes and Regulation Mechanisms

Abiotic stresses such as drought induce the expression of a large number of genes through complex transcriptional regulation. Many related genes have been identified as candidates for drought stress tolerance in studies using genome annotation, functional genomics, and molecular biology in recent years (Table 2). In rice, some such genes have been studied by suppression and overexpression in vivo. Members of transcription factor families such as bZIP, ERF, WRKY, and NAC play key roles in the transmission and response of drought stress signals. For example, OsbZIP23 is a member of the bZIP transcription factor family and upregulates many abiotic stress-related genes through ABA-dependent regulatory pathways. Overexpression of *OsbZIP23* increases ABA sensitivity and improves drought resistance (Zong et al. 2016). Constitutive activation of the transcription factor *OsbZIP46* has also been reported to improve drought tolerance (Tang et al. 2012). DST, a zinc finger transcription factor which is the negative regulator of rice drought resistance, upregulates ROS-related genes and ultimately affects the drought resistance of rice. (Cui et al. 2015). The root system is the first perceiver of soil drought in rice. High expression levels of *DRO1* under drought stress can increase the angle of root growth to a more vertical direction (which is more conducive to water absorption), improving the ability to resist drought stress (Uga et al. 2013). This drought resistance gene has been cloned based on map-based cloning technology.

The plant surface is covered by an epithelial waxy layer, which is the main barrier preventing water loss. At present, some genes related to wax synthesis have been cloned, including those in the *OsGL1* family. Overexpressing these genes thickens the cuticle, affecting the gain and loss of leaf water (Islam et al. 2009). Late embryogenesis abundant (LEA) proteins are important stress-inducible proteins that function in protecting plants against stresses. Transgenic rice plants overexpressing *OsLEA3-1* show enhanced tolerance to drought (Xiao et al. 2007). The gene *LP2* plays a role as a negative regulator of drought response by adjusting stomatal density and closure in ROS metabolism pathways (Wu et al. 2015).

**Table 2:** Genes involved in drought stress

Category	Gene	Accession number	Gene product	Functional role	References
Transcription factors	*OsDREB1F*	LOC_Os01g73770	AP2/EREBP transcription factor	Regulates the ABA-dependent signaling pathway and provides osmotic-stress tolerance	(Wang et al. 2008)
	*OsDREB2B*	LOC_Os05g27930	AP2/EREBP transcription factor	Controls drought stress-induced gene expression through ABA-independent pathways	(Chen et al. 2008)
	*OsNAC5*	LOC_Os11g08210	NAC domain transcription factor	Overexpression significantly thickens roots, improving drought resistance and yield	(Jeong et al. 2013)
	*OsbZIP23*	LOC_Os02g52780	bZIP transcription factor	Regulates the expression of many stress-related genes under abiotic stress through ABA-dependent regulatory pathways, increasing sensitivity to ABA and enhancing drought tolerance	(Song et al. 2020)
	*SNAC1*	LOC_Os03g60080	NAC transcription factor	Increases drought tolerance in transgenic plants (may be due to stomata closure and sensitivity to abscisic acid)	(Hu et al. 2006)
	*OsbZIP12*	LOC_Os01g64730	bZIP transcription factor	Positive regulator of ABA signaling pathway and drought tolerance in rice; may be involved in stress, hormone, and sugar signaling pathways	(Joo et al. 2014)
	*OsLG3*	LOC_Os03g08470	ERF family transcription factor	Regulates drought tolerance by inducing the elimination of ROS	(Joo et al. 2014)
	*OsMYB48-1*	LOC_Os01g74410.2	MYB-type transcription factor	Plays a positive role in drought and salinity tolerance by regulating stress-induced ABA synthesis	(Xiong et al. 2014)
	*OsERF71*	LOC_Os06g09390	Transcription factor in the AP2/ERF family	Promotes drought stress tolerance by increasing expression of genes associated with ABA signaling and proline biosynthesis under stress; recruits factors involved in cell wall modification to enable root morphological adaptations	(Lee et al. 2016; Li et al. 2018)
Others	*DRO1*	LOC_Os09g26840	Auxin response protein	Changes the morphology of the root system, improving the ability of rice to avoid drought conditions	(Uga et al. 2013)
	*DsM1*	LOC_Os02g50970	Mitogen-activated protein kinase kinase kinase	Controls the elimination of ROS by regulating expression of POD genes, thereby regulating drought resistance	(Ning et al. 2010)
	*OsPYL5*	LOC_Os05g12260	Rice orthologue of the ABA receptor	Enhances drought resistance in transgenic lines (related to enhanced stomata closure)	(Kim et al. 2014)
	*OsGL1-2*	LOC_Os02g08230	Glossy1-homologous protein	Affects the loss of leaf water by controlling leaf cuticle wax content, strongly affecting drought resistance	(Islam et al. 2009)
	*LP2*	LOC_Os02g40240	Leucine-rich repeat receptor kinase	Acts as a negative regulator of drought response by regulating ROS metabolism, stomatal density, and stomatal closure	(Wu et al. 2015)
	*OsLEA3-1*	LOC_Os05g46480	Late embryogenesis abundant protein	Overexpression significantly enhances drought tolerance but does not decrease yield	(Xiao et al. 2007)
	*DST*	LOC_Os03g57240	Zinc finger protein	Regulates expression of ROS-related genes and affects ROS accumulation, regulating the opening of stomata and ultimately affecting drought tolerance	(Cui et al. 2015)
	*OsDT11*	LOC_Os11g10590	Alba protein	Mediates drought tolerance in rice (may depend on ABA signaling pathway)	(Li et al. 2017)
	*OsSAP1*	LOC_Os09g31200	Zinc finger protein	Interacts with the transaminase OsAMTR1 and the disease-related protein OsSCP to regulate expression of downstream stress response genes	(Kothari et al. 2016)
	*HYR*	LOC_Os03g02650	Ethylene response factor	Increases rice yield by influencing “morphological-physiological processes” under drought stress conditions	(Ambavaram et al. 2014)
	*OsMADS26*	LOC_Os08g02070	MADS-Box protein	Acts as an upstream regulator of stress-related genes. It is the regulatory center of rice responses to multiple stresses and negatively regulates drought tolerance	(Khong et al. 2015)
	*OsMSR15*	LOC_Os03g41390	C2H2 type zinc finger protein	Regulator of plant response to drought stress	(Khong et al. 2015)
	*OsiSAP7*	LOC_Os03g57900	E3 ubiquitin ligase	Acts as an E3 ubiquitin ligase and functions as a negative regulator for ABA and water deficit stress signals	(Sharma et al. 2015)
	*OsSRO1c*	LOC_Os03g12820	Similar to radical-induced cell death one (SRO) protein	Stimulates stomata closure and H_2_O_2_ accumulation through a novel pathway containing SNAC1 and DST	(You et al. 2013)
	*OsNCED2*	LOC_Os12g24800	9-cis-epoxycarotenoid dioxygenase	Plays key roles in the ABA synthesis pathway and contributes to aerobic adaptation of upland rice	(Li et al. 2021)
	*OsGUDK*	LOC_Os03g08170	Receptor-like kinase	Mediates drought stress signaling through phosphorylation and activation of OsAP37, resulting in transcriptional activation of stress-regulated genes, imparting tolerance and improving yield under drought stress	(Ramegowda et al. 2014)
	*OsGhd2*	LOC_Os02g49880	CONSTANS (CO)-like genes	Controls grain number, heading date, and plant height; positively regulates drought stress-triggered early senescence in rice	(Liu et al. 2016a)

## Responses to Combined Heat and Drought Stress

Heat and drought stress are typical abiotic stresses from which crops often suffer due to climate change (Mizoi et al. 2013). These environmental factors may have tremendous impacts on yield, depending on the crop variety, plant growth stage, and the intensity and duration of stress. Heat and drought stress frequently co-occur across hot tropical regions. Rice cultivar N22 displays heat and drought tolerance as a result of better anther dehiscence, pollen number, and pollen viability (Fahad et al. 2018). A temperature of 36.5 °C is vital limit for such tolerance during flowing stage (Bahuguna et al. 2015). In the summer, temperatures often surpass this point, leading to associated water deficit (Bahuguna et al. 2015). Plants generally close their stomata in response to rapid water loss from plant tissues or the soil, but this strategy may cause increased tissue temperatures as a result of impaired transpiration for cooling. Furthermore, high temperatures can result in drought stress through evapotranspiration. Thus, high temperature and water deficit represent a combined stress that elicits special physiological and molecular behaviors (Jin et al. 2016).

**Fig. 1:**
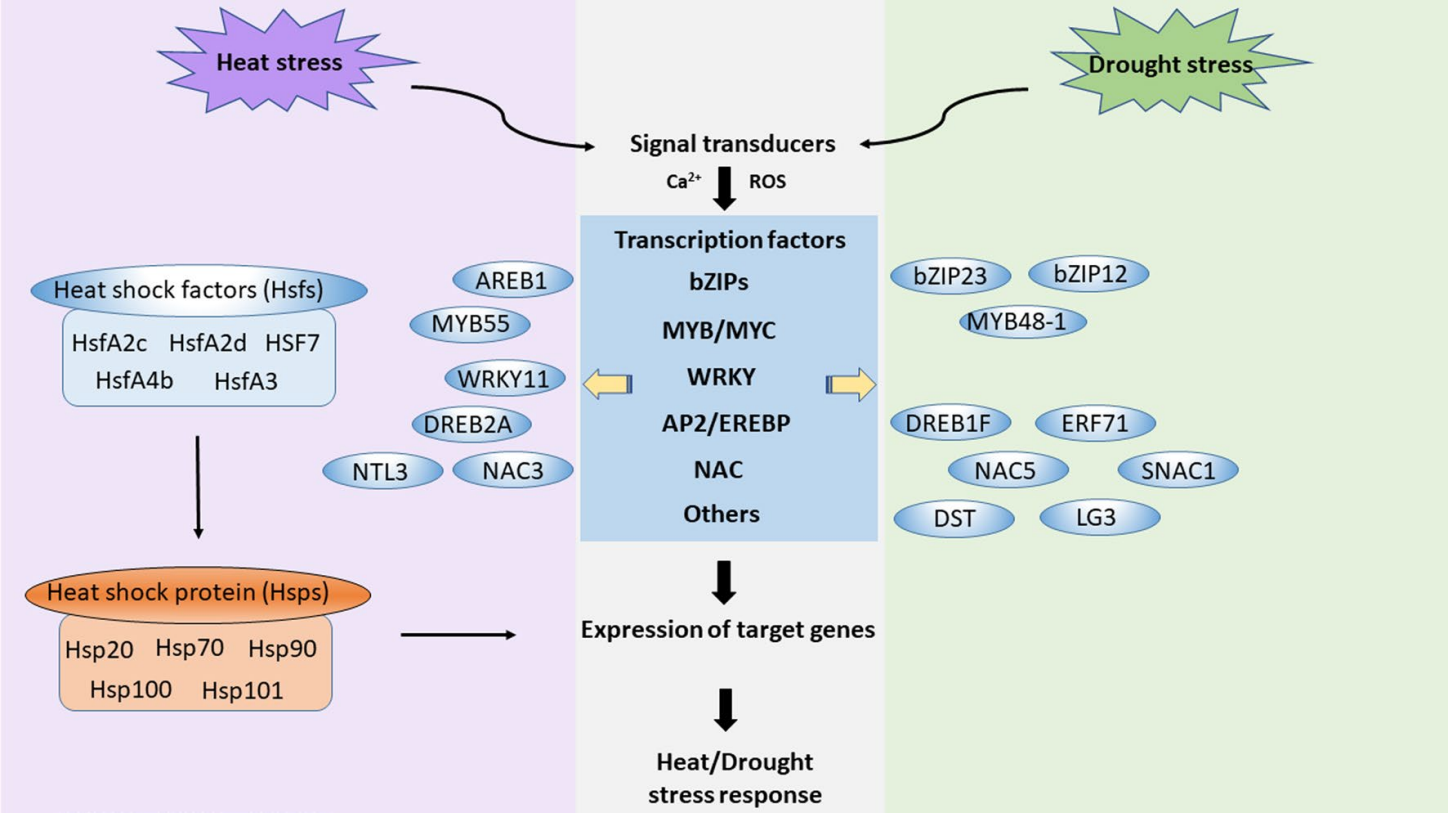
A genetic network diagram of drought and heat stresses in rice

There are many similarities in the effects of drought and high temperature on rice physiology and growth. The impact of drought and heat stress have been extensively studied in isolation, whereas these abiotic stresses exist in combination more frequently in the field (Farooq et al. 2012). There are many aspects in common with respect to genes and genetic regulation mechanisms involved in the rice response to drought and heat stress. For example, a combination of drought and heat stress induces genes encoding CAT, pathogenesis-related proteins, and WRKY transcription factors, and suppresses genes encoding POD and photosynthetic proteins. The co-occurrence of both high temperature and drought has been shown to considerably increase the negative effects on plants. It results in increased respiration and reduced photosynthesis, stomatal conductance, leaf area, and water-use efficiency (Shah and Paulsen 2003). Exposure to both stresses in combination can cause the production of ROS, further triggering protective responses such as increasing the expression and activity of ROS-scavenging enzymes and molecules. At the flowering stage, the combined stress results in reduced fertilization due to impaired ovule function and has negative impacts on pollen development, including sterility (Prasad et al. 2008). A genetic network diagram of drought and heat stresses is shown in Fig. 1. Upon heat/drought stress, rice senses the stress signals through signal transducers including Ca^2+^ and ROS. The transcription factors are activated. Both heat stress and drought stress induce transcription factors of bZIPs, MYB/MYC, WRKY, AP2/EREBP, NAC and so on. Different transcription factors can be triggered individually in heat stress or drought stress. For example, for heat stress, the specific Hsfs can be induced and they activate the downstream Hsps. Then the signals initiate expression of target genes and the following heat/drought response. The knowledge gained over the past several decades has contributed to understanding of key mechanisms involved in plant abiotic stress tolerance, but many mechanisms remain unclear.

## The Genetic Improvement of New Heat- and Drought-Tolerant Varieties

### Breeding New Heat- and Drought-Tolerant Varieties

The ability of different rice varieties to tolerate heat and drought varies greatly. The selection of high-temperature and drought-resistant varieties can reduce the damage caused by high temperature or drought to a certain extent. The genetic improvement of complex traits, especially tolerance of environmental stresses such as temperature and drought, is a very challenging task for rice breeders (Langridge and Reynolds 2015). Such traits have complex gene regulatory networks, and it is therefore necessary to comprehensively consider a variety of factors when breeding heat- and drought-tolerant varieties. Combining traditional breeding techniques with genomic information can aid in efficient improvements in rice heat and drought tolerance.

Molecular marker technology allows researchers to characterize a huge number of germplasm resources at the molecular level, evaluate the diversity of heat- and drought-tolerant germplasm, and identify the best heat- and drought-tolerant resources for breeding use (Mishra et al. 2018). Although rice is in general highly sensitive to heat and drought stress, the extensive genetic diversity among rice varieties, subspecies, and wild relatives provides a vast amount of available allelic variation for heat and drought tolerance studies (Lafitte et al. 2006). The use of molecular markers to analyze the evolutionary relationships and systematic classification of germplasm has accelerated the exploration and development of natural allelic variation, thereby improving heat and drought tolerance of rice. An analysis of the genotypic diversity of 46 indica rice varieties found extensive variation and identified two highly drought-tolerant lines (Norungan and TK-M1) (Vivek et al. 2004).

The construction of a high-density quantitative trait locus (QTL) map for heat and drought resistance traits in rice could provide a strong foundation for molecular marker-assisted breeding. With the help of molecular markers to accurately locate QTLs related to heat and drought resistance traits, these QTLs can be efficiently grouped and cultivated more efficiently to produce heat and drought-resistant rice varieties. The heat-resistant rice strain HT54 and the heat-sensitive strain HT13 were used to construct a mapping population and located *OsHTAS*, the major site of heat tolerance regulation in the seedling stage of HT54 (Wei et al. 2013). Further research found that the *OsHTAS* gene encodes a ubiquitin ligase that improves heat tolerance by regulating the closure of leaf stomata induced by H_2_O_2_ under heat stress (Liu et al. 2016b). qDTY 4.1 was introduced into IR64 rice variety through molecular markers (Kumar et al. 2014). The results of the study proved that under severe drought conditions during the reproductive period, the yield advantage of IR64 near isogenic lines (NILs) was more than 100% higher than that of the parent IR64.

Genetic engineering can be used to improve plant stress resistance by changing one or several genes. It has become an important method for studying stress resistance of plants and will greatly accelerate the efficiency of breeding. Genetic engineering technology can not only be used to study endogenous rice genes, but also the effects of exogenous genes from other species; this is a key advantage over traditional or marker-assisted breeding (Cattivelli et al. 2008). In recent years, significant progress has been made in transgenic rice developed by reproducible *Agrobacterium*-mediated gene transfer and gene editing technology. Many genes involved in heat and drought stress responses (signal transduction, post-translational modification, and metabolite production) have been introduced into rice to verify their functions.

Genome editing is a breakthrough technology in the field of life sciences that has been developed in recent years (Roy et al. 2021). It can be used to accurately modify a target gene without changing the overall stability of the genome, and the final product is free of any foreign DNA. Especially in the past several years, the rapid development of precision gene editing technology has resulted in faster and more effective breeding methods for rice. At present, the precise genome editing systems in plants mainly include deaminase-mediated base editing technology and reverse transcriptase-mediated lead editing systems. Several studies have reported the use of CRISPR-Cas to improve crop characteristics including yield, quality, disease resistance, and herbicide resistance (Zhu et al. 2020). Genome editing technology has broad application prospects, such as providing genetic resources for molecular breeding of heat- and drought-tolerant rice varieties (Chennakesavulu et al. 2021).

Breeding new tolerant varieties is often based on genetic mechanisms. Take the heat-tolerant gene *TT1* for example, it protects cells from heat stress by degradation of harmful ubiquitinated proteins (Li et al. 2015b). Generally, under the condition of high temperature, the protein in the cell will lose a lot of activity and become toxic, causing the rice to wither and die. The rice with the introduction of the TT1 gene can quickly degrade the proteins denatured by high temperature, and remove these toxic wastes to avoid the death of the rice. The study also explored the role of *TT1* in the response to high temperature of turfgrass and *Arabidopsis*, showing that it has a function of improving plant high temperature tolerance in different species. These findings also suggest that *TT1* has broad application potential in high temperature resistance breeding of grass crops including wheat and cruciferous vegetables such as Chinese cabbage. In subsequent studies, another heat-resistant QTL locus, *TT2*, from tropical japonica was obtained (Kan et al. 2022). When *TT2* function is lost, the heat-induced calcium signal is weakened, which in turn weakens the interaction of related proteins, reduces the inhibition of transcriptional activity, and finally maintains the normal expression of related protein and stable wax content under high temperature stress, thus showing a heat resistant phenotype. Through backcrossing, the researchers successfully introduced it into the high-quality Guangdong rice variety “Huajingxian 74”, thereby cultivating a new heat-resistant line with heat-resistant loci. Compared with the backcross parent Huajing Indica 74, the survival rate of this line at the seedling stage was increased by 8 to 10 times. At the same time, the introduction of this locus also enhanced the heat resistance at the mature stage, which was mainly manifested as a single plant under high temperature stress. Production increased by 54.7%. In addition, heat-resistant strains can also be obtained by targeted gene knockout of *TT1* in wheat, maize and other crops, thereby greatly shortening the breeding cycle. Precious genetic resources such as the mentioned genes can be used for crop heat resistance breeding, which is of great significance for the future targeted heat resistance genetic improvement of crops by means of molecular design.

### Improvement in Field Management

Farming and cultivation techniques to reduce the damage caused by high temperature and drought are the main measures used in rice production to minimize effects on crop yield and quality (Korres et al. 2017). For example, based on the time periods in which rice is most sensitive to high temperature and drought, the sowing period can be adjusted to avoid these stressors during the flowering and fruiting period; this has become one of the primary countermeasures used to alleviate the effects of high temperature and drought during the flowering period (Wang et al. 2019).

Drought-resistant cultivation technology is also an important method to deal with drought (Luo 2010). Rice with well-managed field production has a stronger ability to defend against stress. Measures to improve drought tolerance include timely irrigation, reasonable fertilizer application during the seedling stage, and timely drying of the field during the tillering stage to reduce ineffective tillers (Luo et al. 2019). Dry direct seeding of rice is a simple cultivation technique that can be adapted to rice planting in arid and rainless areas while alleviating labor tension. Compared with traditional rice cultivation, dry direct seeding technology eliminates raising seedlings and transplanting, saves production costs, and conserves irrigation water. From this perspective, the strategy has a bright future. Managing the relationship between rice growth and water supply at each growth stage, and adopting a combination of cultivation and irrigation control measures, can increase the water-saving capacity of rice (Tuong 2000). Studies have shown that shallow water irrigation can be applied after rice transplanting to the effective tillering stage, jointing and booting stage, and heading and flowering stage. Moisture irrigation is adopted for other growth stages, and water will dry out naturally at the maturity stage. This alternate dry and wet irrigation can save water by 19–39%, and has little effect on output (Belder et al. 2004). Nitrogen also has an important influence on the yield and quality of rice (Chaturvedi 2005). Under high temperature stress, the application of nitrogen panicle fertilizer (medium and high nitrogen) during panicle differentiation can significantly increase seed setting rate, 1000-grain weight, and yield. Scientific fertilization is conducive to the formation of a good population structure, and furthermore reduces panicle and canopy leaf temperature and increases the resistance of rice plants to high temperatures. In summary, in rice cultivation, different levels of water and fertilizer application can be optimally timed to save water, stabilize yield, and improve grain quality (Kakar et al. 2020).

### Chemical Control Technology

**Fig. 2:**
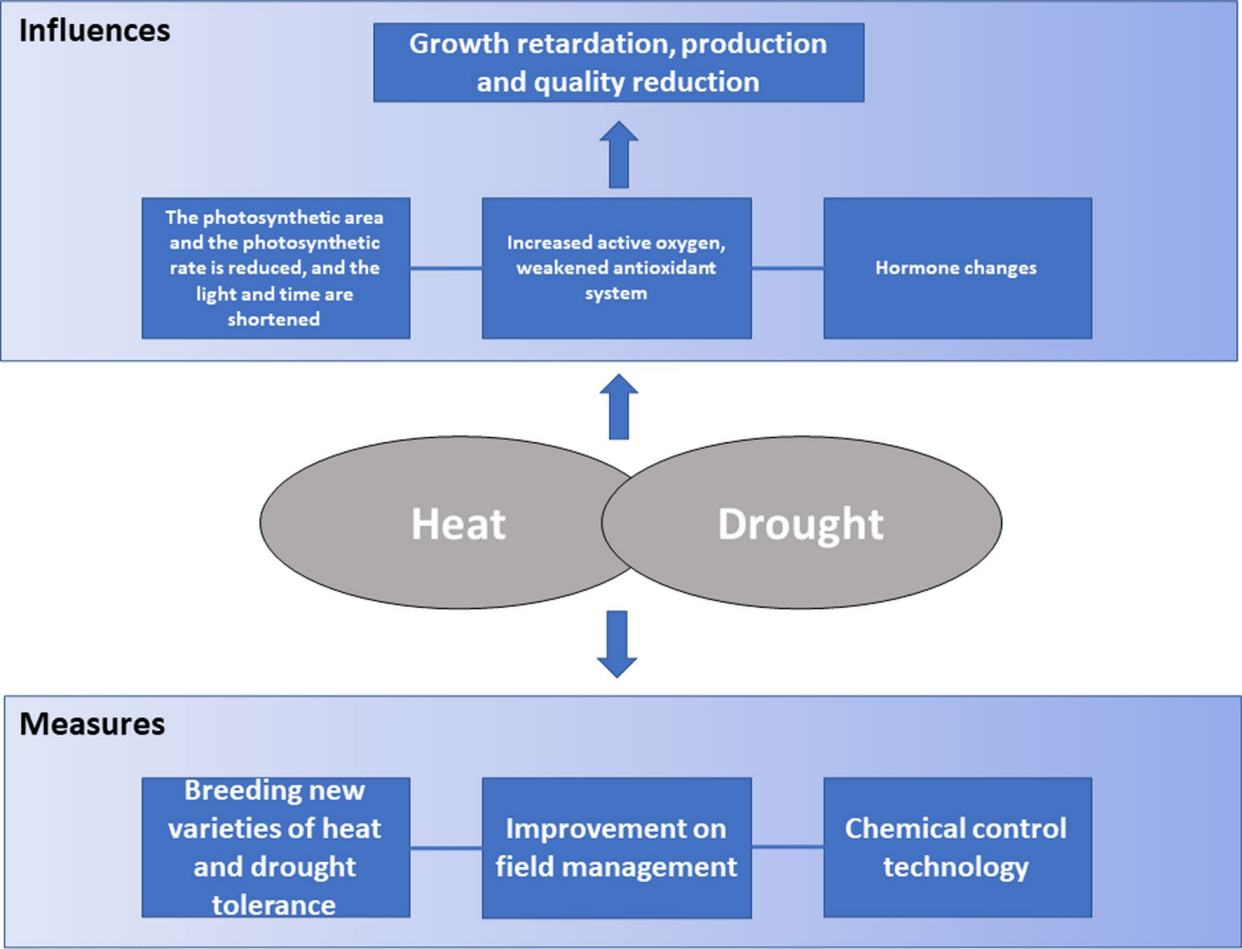
A brief overview of the influences of heat and drought stress on rice and known countermeasures

Under high temperature conditions, application of exogenous substances such as extra-root fertilization on the surface of rice can mitigate the adverse effects of high temperature and reduce heat damage (Fábián et al. 2019; Yang et al. 2021). For example, during the vegetative or flowering period, spraying 0.2% boron fertilizer can increase the activity of antioxidant enzymes, increase membrane stability and sugar transport, and improve pollen vigor, spikelet fertility, and rice yield. (Shahid et al. 2018). Spraying brassinolide can increase the synthesis of Hsp (Dhaubhadel et al. 2002). Foliar spraying of Spd in the early stage of rice filling maintains osmotic balance, reduces MDA content, and increases SOD and POD activity, soluble sugar content, and photosynthetic and transpiration rates (Tang et al. 2018). The combined application of four plant growth regulators (ASA, tocopherol, brassinosteroid, and methyl jasmonate) promotes rice photosynthesis and grain filling, improves spikelet fertility, and effectively alleviates high temperature damage (Fahad et al. 2016b; Fahad et al. 2016a). Research found through seed soaking and foliar spraying of Put, Spd, and Spm that the use of PAs can increase the production of free proline, cyanocyanidin, and soluble phenols, reduce ROS damage to cell membranes, and improve the water utilization rate (Farooq et al. 2009). Spraying of growth regulators such as salicylic acid, brassinolide, and PAs can alleviate the damage caused by drought, but the production costs of most chemical control agents are too high and are not suitable for large-scale agricultural production (Ashraf et al. 2011). Acetate has a significant role in orchestrating plants’ survival capability and enhancing drought tolerance (Kim et al. 2017). In the future, it will be necessary to select cultivation control measures that promote physiological regulation of crops, i.e., that create an environment for the crop to utilize its own resistance and tolerance mechanisms (Zhang et al. 2021). The influences of heat and drought stress on rice and countermeasures are summarized in Fig. 2.

## Conclusion

Heat and drought stress are two typical abiotic stresses that plants frequently encounter in the natural environment. They have adverse impacts on rice growth and development, yield and quality, and physiological and biochemical characteristics. Heat and drought tolerance responses are mediated by a series of complicated signaling pathways. These signaling mechanisms include ion transporters, free radical scavengers, signaling cascades, and transcriptional control of a series of proteins and elements. The coordinated operation of these components is necessary to tolerate stress. Transcription factors are key players in heat- and drought-resistance signal transduction; they are continuously synthesized during the stress response to function in signal transmission and amplification to regulate the expression of downstream genes, ultimately causing a series of plant resistance responses. Heat and drought resistance in rice involve the expression and regulation of a large number of genes, many of which are known to protect cells from the adverse effects of stressors. The current review summarizes heat- and drought-stress genes, their functioning mechanisms in stress responses and the application in breeding. It implies how to make use of genetic mechanisms and connecting it to breeding by various genetic manipulation approaches. The molecular characterization of genes/pathways related to heat and drought tolerance and improving the creation of stress-tolerant materials will be of great value for food production. Future work should focus on screening heat- and drought-resistant varieties, extensively collecting tolerant rice germplasm resources, and selecting new resistant and high-yield rice varieties.

## Data Availability

Data sharing is not applicable to this article as no datasets were generated during this study.

## Abbreviations

ABA: Abscisic acid
ASA: Ascorbic acid
CAT: Catalase
CRISPR: Clustered regularly interspersed short palindromic repeats
CTK: Cytokinin
ER: Endoplasmic reticulum
GA: Gibberellic acid
GR: Glutathione reductase
GSH: Glutathione
Hsfs: Heat shock factors
Hsps: Heat shock proteins
IAA: Indoleacetic acid
MDA: Malondialdehyde
NILs: Near isogenic lines
PAs: Polyamines
POD: Peroxidase
Pro: Proline
PSI: Photosystem I
PSII: Photosystem II
Put: Putrescine
QTL: Quantitative trait locus
ROS: Reactive oxygen species
SA: Salicylic acid
SOD: superoxide dismutase
Spd: Spermidine
Spm: Spermine
